# FREQUENCY OF SOCIAL MEDIA USE IS ASSOCIATED WITH DELAY DISCAOUNTING IN OLDER ADULTS

**DOI:** 10.1093/geroni/igae098.4195

**Published:** 2024-12-31

**Authors:** Allison Walton, Daniel Moss, Abigail Stephan, Lesley Ross, Christine Phillips

**Affiliations:** Clemson Institute for Engaged Aging, Seneca, South Carolina, United States; Clemson University Institute for Engaged Aging, Seneca, South Carolina, United States; Clemson University, Clemson, South Carolina, United States; Clemson University, Clemson, South Carolina, United States; Clemson University Institute for Engaged Aging, Seneca, South Carolina, United States

## Abstract

Delay discounting is the decline in the perceived value of a reward as the time to reward receipt increases. Individuals who discount more steeply favor smaller more immediate rewards (i.e., impulsive choices) over larger delayed rewards (i.e., self-controlled choices). Social media and smartphone engagement, whereby users have instant access to information or social networks, are linked with higher delay discount rates in younger adults. However, there is a dearth of research surrounding this relationship in older adults. The present study examined associations between delay discounting, use of health and financial tracking apps, and social media use, as well as age as a potential moderator, in community-dwelling adults ages 55 years and older (M=68.64, SD=8.2, n=120) enrolled in the Tracking Real-world Activities in Life Study. Delay discount rate was assessed using the Monetary Choice Questionnaire. Three self-report items assessed frequency of app and social media use. Multiple linear regression results indicated delay discount rate was positively associated only with social media use, statistically controlling for age (β =.17, t(118) = 2.14, p =.035). Age did not moderate this relationship. Results suggest that regardless of age, more frequent social media use is linked with greater preference for immediate rewards. Future research should explore whether frequent engagement with social media increases delay discount rate or if those who discount more steeply are more inclined to engage with social media. Additionally, potential interventions targeting impulsivity and delay discounting are needed in this population who are at risk of online scams

